# The role of landscape in shaping bird community and implications for landscape management at Nanjing Lukou International Airport

**DOI:** 10.1002/ece3.9646

**Published:** 2023-01-06

**Authors:** Sijia Yuan, Keer Miao, Ruen Qian, Yang Zhao, Dongfang Hu, Chaochao Hu, Qing Chang

**Affiliations:** ^1^ Jiangsu Key Laboratory for Biodiversity and Biotechnology, School of Life Sciences Nanjing Normal University Nanjing China; ^2^ Nanjing Lukou International Airport Nanjing China; ^3^ Analytical and Testing Center Nanjing Normal University Nanjing China

**Keywords:** airport, bird diversity, bird strike prevention, landscape attributes

## Abstract

Understanding the patterns of bird diversity and its driving force is necessary for bird strike prevention. In this study, we investigated the effects of landscape on phylogenetic and functional diversity of bird communities at Nanjing Lukou International Airport (NLIA). Bird identifications and counting of individuals were carried out from November 2017 to October 2019. Based on the land‐cover data, the landscape was divided into four main types, including farmlands, woodlands, wetlands, and urban areas. Bird phylogenetic and functional diversity were strongly affected by landscape matrix types. Species richness and Faith's phylogenetic distance were highest in woodlands, while mean pairwise distance (MPD), mean nearest‐taxon distance (MNTD), and functional dispersion (FDis) were highest in wetlands. Based on the feeding behavior, carnivorous birds had the lowest species richness but had the highest FDis, which implied that carnivorous birds occupied most niches at the NLIA. Moreover, bird assemblages exhibited phylogenetic and functional clustering in the four kinds of landscapes. A variety of landscape attributes had significant effects on species diversity, phylogenetic and functional diversity. Landscape‐scale factors played an important role in the shaping of bird communities around NLIA. Our results suggest that landscape management surrounding airports can provide new approaches for policymakers to mitigate wildlife strikes.

## Graphical abstract

Bird multi‐dimensional diversity were strongly affected by landscape matrix types. Landscape played an important role in shaping bird communities. Edge density, shape complexity and area were the relatively important explanatory factors. Our results suggest that landscape management surrounding airports can provide new approaches for policymakers to mitigate wildlife strikes

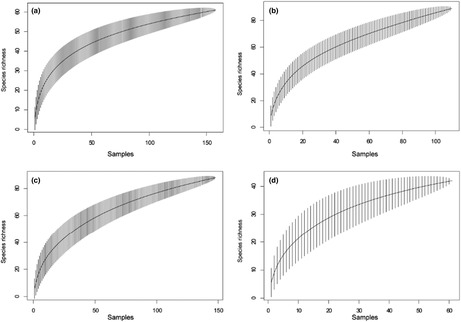

## 1 INTRODUCTION

Understanding species diversity patterns is helpful to determine strategies for conservation (Li et al., 2019). Birds are an important component of the ecosystem, and are considered indicator species for habitat quality because of their sensitivity to changes in habitat structure (Kirk et al., 2020; Moning & Müller, 2008). Previous studies have demonstrated that bird communities in agricultural and grassland landscapes have substantially declined due to habitat loss (Fischer et al., 2011; Pavlacky et al., 2022). Moreover, birds can respond to urbanization gradients worldwide by changing species richness, abundance, and composition (Filloy et al., 2019). Therefore, bird diversity is a valuable guide for conservation management at regional and landscape levels.

With human population growth, the majority of the land surface has been converted into a human‐developed landscape (Pfeiffer et al., 2018). This continuous urban development leads to the fragmentation, isolation, and degradation of natural habitats, and is accompanied by severe impacts on the biotic communities living in urban landscapes (Schütz & Schulze, 2015). Many studies have shown that building cover is detrimental to bird richness and more generalist birds are found in human‐developed habitats (Cresswell et al., 2020; Morelli, Benedetti, Floigl, & Ibáñez‐Álamo, 2021; Morelli, Benedetti, Ibáñez‐Álamo, et al., 2021).

One of the important urban landscapes is the airport which has irreplaceable functions in transportation (Pfeiffer et al., 2018). However, bird strikes have been intensive with the development of the aviation industry, causing enormous economic losses worldwide each year (Jeffery & Buschke, 2019). Therefore, there is a specificity to the conservation of bird diversity in the airport area, that is, to seek a balance between reducing bird strikes and conserving biodiversity. Many studies focus on specific bird groups directly linked to the rate of bird strikes (Andersson et al., 2017; DeVault et al., 2016). Compared with songbirds, waterfowls such as ducks and geese cause more damages to aircraft (Andersson et al., 2017), with their density, body mass, and group size significantly influencing the likelihood of aircraft damage (DeVault et al., 2016). Raptors also account for a high of bird strikes (Blackwell & Wright, 2006).

However, the diversity of bird communities in the areas surrounding airports has rarely been studied, although airports have been certified to preserve a high level of biodiversity (Blackwell et al., 2013). Several studies conducted in China indicate differences in bird diversity across landscapes, thus classified ecological environment management can be implemented to prevent bird strikes (Liu et al., 2021; Wu et al., 2015). Moreover, these studies merely focus on taxonomic diversity, without consideration of phylogenetic and functional diversity which have been studied in other landscapes (Frishkoff et al., 2014; Jia et al., 2020). Phylogenetic and functional diversity can provide information about bird phylogeny and functional traits, respectively, which help better characterize the community (Barbaro et al., 2014; Winter et al., 2013). It is widely recognized that phylogenetic and functional perspectives can be useful to disentangle the role of ecological processes (e.g., environmental filtering and competitive exclusion) which govern the assembly of bird communities (Gómez et al., 2010). Integrating phylogenetic and functional diversity is increasingly considered in wildlife management and conservation planning (Dehling et al., 2022; Winter et al., 2013). Evidence of biological invasions suggests the positive association of taxonomic, phylogenetic, and functional diversity with bird species richness, emphasizing the importance of considering different facets of biodiversity in wildlife management (Andrikou‐Charitidou et al., 2020). A study conducted in protected areas in Spain indicates that taxonomic, functional, and phylogenetic diversity show differences among environment types, which suggests the importance of considering different facets of biodiversity simultaneously for a better spatial prioritization (Morelli, Benedetti, Floigl, & Ibáñez‐Álamo, 2021; Morelli, Benedetti, Ibáñez‐Álamo, et al., 2021). These experiences can be used as a guide to study the pattern of bird diversity in different matrix types in and around the airport.

Many studies concentrate on the primary variables underlying biodiversity near the airport in order to prevent bird strikes (Conkling et al., 2018). Biotic or abiotic factors, for instance, crop types, vegetation composition, food availability, and landscape structure, are proven to affect community composition at the airport (Alquezar et al., 2020; Iglay et al., 2017; Pennell et al., 2016). Among the factors mentioned above, landscape structure is relatively less studied in the airport area. Most of the studies consider the effects of land use on biodiversity surrounding airports (Alquezar et al., 2020; Fox et al., 2013). Only a few studies indicate that the strike rate is positively influenced by large areas of wetlands, close proximity of wetlands, and landscape diversity at different extents from airports (Pfeiffer et al., 2018, 2020). Fragmentation effects on some urban birds are linked to the type of peri‐urban matrix (Hedblom & Söderström, 2010). Studies have shown that the proportion of developed land and forest edge density of cities have consistent negative effects on bird richness in urban landscapes (Filloy et al., 2019; Soifer et al., 2021). However, the effects of landscape attributes on bird diversity surrounding the airport remain poorly known. For the development of efficient and informed biodiversity policies to reduce the rate of bird strikes, it is essential to understand how landscape attributes affect biodiversity patterns on the landscape scale (Pfeiffer et al., 2020; Rüdisser et al., 2015).

In this study, we focused on the phylogenetic and functional diversity of bird communities and functional groups at Nanjing Lukou International Airport (NLIA), which is located in Jiangsu Province, China. We divided land‐cover types into four main landscape matrix types (farmlands, woodlands, wetlands, and urban areas) and extracted landscape attributes of class level and landscape level to evaluate their effects on phylogenetic and functional richness and structure of bird assemblages. Here, we aim to address the following issues: (1) what is the pattern of bird diversity in different matrix types; (2) what is the pattern of bird functional groups' diversity; and (3) whether bird diversity and community structure are influenced by landscape attributes.

## 2 METHODS

### 2.1 Study area

**FIGURE 1:**
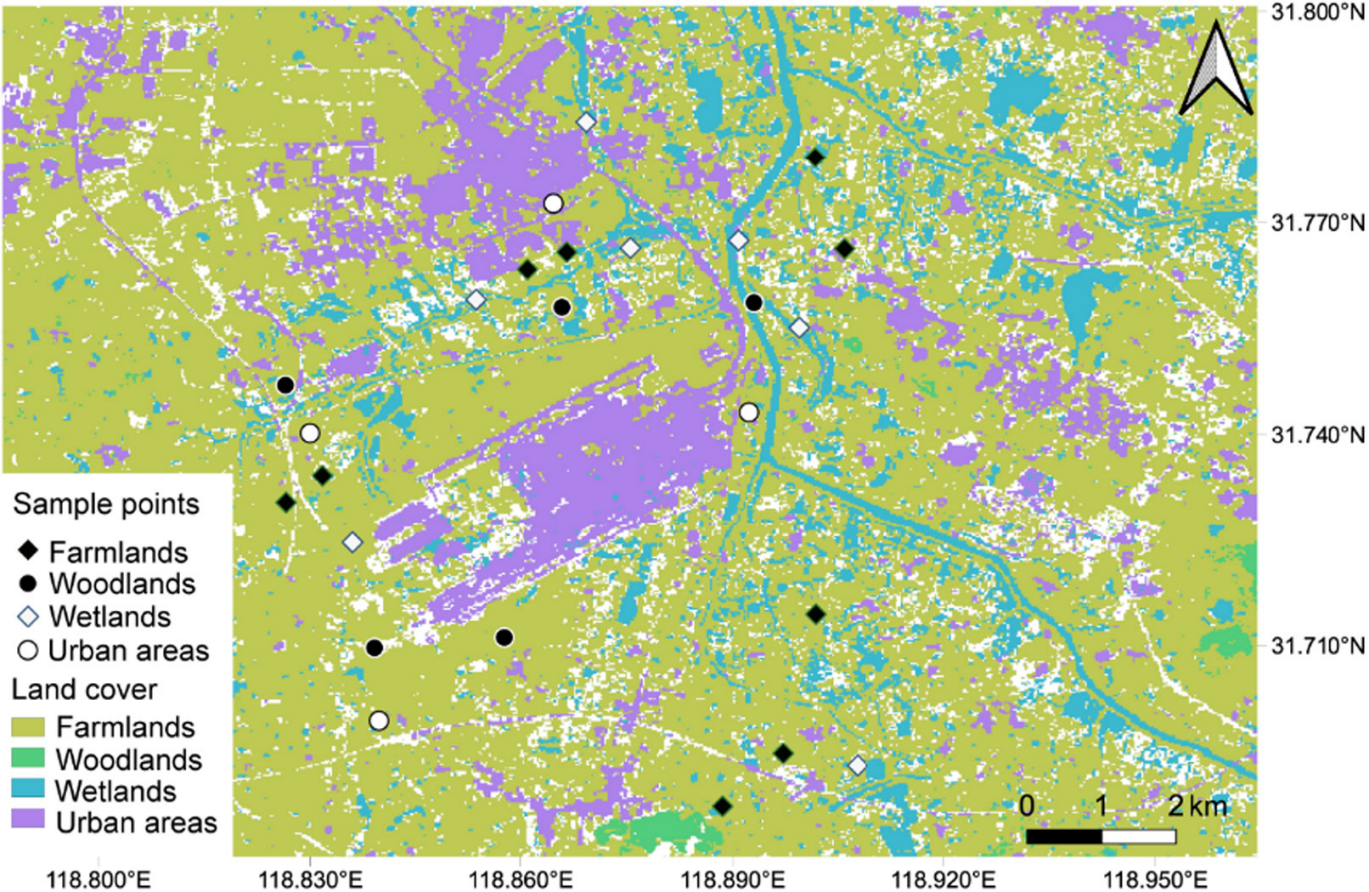
The location of the sampling points and landscape types at Nanjing Lukou International Airport (NLIA).

We conducted the study within 8 km radius extent from the center of Nanjing Lukou International Airport (31°30′–31°56′N, 118°37′–119°60′E). The region experiences a subtropical monsoon climate with an annual mean temperature of 15.4°C and annual mean precipitation of 1106 mm. Land‐cover data were obtained to evaluate the effects of landscape matrix types, which were downloaded from the Yangtze River Delta Science Data Center, National Earth System Science Data Center, National Science & Technology Infrastructure of China (http://nnu.geodata.cn:8008), with a resolution of 9 × 9 m per grid cell. We totally extracted 19 land‐cover types through image interpretation in QGIS 3.16.9 (QGIS Development Team, 2021). Considering the relatively low proportion of certain land‐cover types, we integrated the 19 land‐cover types into four main matrix types: farmlands, woodlands, wetlands, and urban areas, with their areas accounting for 69.66%, 15.05%, 8.42%, and 6.87%, respectively (Figure 1). These matrix types are the most continuous and widely distributed landscape structures and are often considered in birds and landscape studies (Filloy et al., 2019; Fischer et al., 2011; Hedblom & Söderström, 2010; Soifer et al., 2021).

### 2.2 Bird survey

**FIGURE A1:**
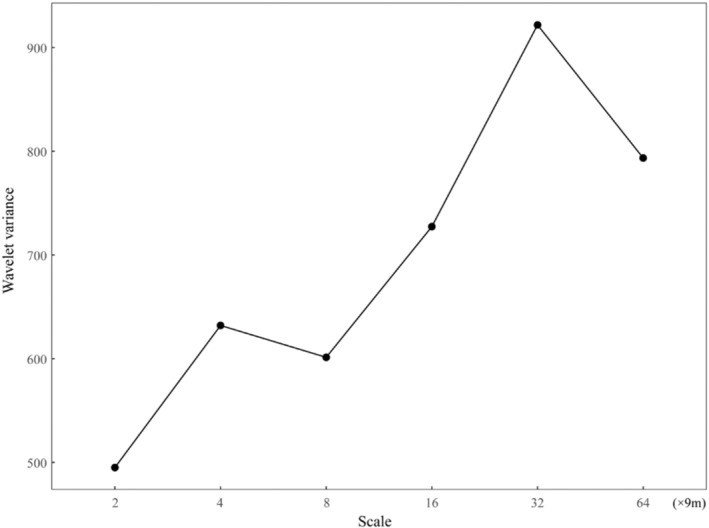
Wavelet variances under different scales within 8 km radius extent from Nanjing Lukou International Airport (NLIA). Scales were represented by power 2 mode multiplied by resolution of imagery data (9 m).

Wavelet analysis, a spectral decomposition technique, was used to determine the key spatial scale that was important to quantify the relative influence of matrix types on species distributions (Fortin et al., 2012). We generated 1000 random points within 8 km radius extent from the NLIA and extracted their land‐cover types. Then, we ran the wavelet analysis with MATLAB 9.9 (MathWorks, Natick, Massachusetts; https://ww2.mathworks.cn/products/matlab.html). The result showed that a 288‐m radius from the center of each point was appropriate (Figure A1). We then randomly selected 9 sampling points in farmlands, 7 in wetlands, 5 in woodlands, and 4 in urban areas (25 points in total) to make sure that sampling effort on each matrix type was roughly proportional to its area (Figure 1). Sampling points were at least 576 m apart. From November 2017 to October 2019, we carried out point‐count bird surveys monthly (Sorace et al., 2000). Each observation was in 20 min. We usually surveyed birds on sunny and windless days. We recorded all birds seen or heard and excluded flyovers to obtain the birds' data.

Considering that the combination of functional groups and landscape matrices enables a comprehensive assessment of bird diversity (French & Picozzi, 2002), we chose three main functional groups (i.e., carnivorous birds, insectivorous birds, and omnivorous birds) based on the traits data collected by Wang et al., 2021 (Table A1). The species accumulation curve (SAC) is a useful tool to determine the validity and adequacy of sampling (Ugland et al., 2003). We used R package “vegan” (Oksanen et al., 2013) to plot species' accumulation curve for bird communities in each matrix type.

### 2.3 Bird phylogeny and functional traits

We cut the global phylogenetic tree of birds by subsampling 5000 “Hackett All species: a set of 10,000 trees with 9993 OTUs each” trees from BirdTree (http://birdtree.org; Jetz et al., 2012). Then, we constructed a new maximum clade credibility tree with a 0.5 posterior probability limit by the software TreeAnnonator v1.10.4 in the BEAST package v1.10.4 (Suchard et al., 2018).

We focused on four kinds of functional traits relevant ecologically to bird strikes: morphological characteristics (body length, bill length, wing length, tarsus length, and body mass), nest sites (ground, water, shrub, canopy, and wall), feeding behaviors (carnivorous, insectivorous, omnivorous, granivorous, and piscivorous), and foraging strata (below water surface, ground, understory, and mid‐high) (Wang et al., 2021; Wilman et al., 2014). Morphological characteristics were continuous values and others were treated as binary traits (Table A2). In this study, we estimated ecological processes of bird community assembly, which depends on the conservatism of functional traits (Srivastava et al., 2012). To test trait conservatism, we calculated phylogenetic signals using Blomberg's *K* for continuous traits (Blomberg et al., 2003) and statistic *D* for binary traits (Fritz & Purvis, 2010) with R packages “phytools” (Revell, 2012) and “caper” (Orme et al., 2013), respectively.

### 2.4 Phylogenetic and functional diversity measures

Based on the new phylogenetic tree constructed, we calculated Faith's phylogenetic distance (Faith's PD) to describe the total sum of phylogenetic history (Faith, 1992). We also calculated mean pairwise distance (MPD) and mean nearest‐taxon distance (MNTD) to represent phylogenetic structure (Webb et al., 2002). We used null models to infer whether communities exhibited phylogenetic clustering or over‐dispersion (Jia et al., 2020). We calculated the standardized effect size (SES) for values of MPD and MNTD based on 999 null models using R package “picante” (Kembel et al., 2010).

We computed functional richness (FRic) and functional dispersion (FDis) to represent functional diversity and structure. Then, we infer whether communities exhibited functional clustering or over‐dispersion in the same way as for phylogenetic diversity. With the species‐by‐trait matrix, we first constructed a functional dendrogram using Gower distance with UPGMA method (Petchey & Gaston, 2002). We computed mean pairwise functional distance (MFD) and mean functional nearest‐taxon distance (FD.MNTD) based on the functional dendrogram. Then, we calculated SES for MFD and FD.MNTD. The value of SES indicates phylogenetic or functional clustering when <0, while over‐dispersion generates values higher than 0 (Si et al., 2017). All metrics were calculated using R package “picante” (Kembel et al., 2010) and “FD” (Laliberté & Legendre, 2010). Species richness (SR), MPD, MNTD, and FDis of functional groups were also calculated to evaluate phylogenetic and functional structure of different functional groups.

### 2.5 Landscape attributes

We calculated landscape attributes of the class level and landscape level to evaluate the effects of matrix types and landscape within 8 km radius extent from the NLIA on bird diversity, respectively. Number of patches (NP), aggregation index (AI), edge density (ED), mean of patch area (AREA‐MN), mean perimeter–area ratio (PARA‐MN), mean fractal dimension index (FRAC‐MN), effective mesh size (MESH), and Shannon's diversity index (SHDI) were selected (Gao et al., 2021; Zhang et al., 2021; Table 1), which were calculated using R package “landscapemetrics” (Hesselbarth et al., 2019).

**TABLE 1:** Summary of the landscape attributes used for investigating the effects of landscape on phylogenetic and functional richness and structure of bird assemblages

Level	Category	Landscape attributes	Abbreviation
Class level	Aggregation metric	Number of patches	NP
		Aggregation index	AI
	Area and edge metric	Edge density	ED
		Mean of patch area	AREA‐MN
	Shape metric	Mean perimeter–area ratio	PARA‐MN
		Mean fractal dimension index	FRAC‐MN
Landscape level	Aggregation metric	Number of patches	NP‐l
		Effective mesh size	MESH
	Area and edge metric	Mean of patch area	AREA‐MN‐l
	Shape metric	Mean perimeter–area ratio	PARA‐MN‐l
		Mean fractal dimension index	FRAC‐MN‐l
	Diversity metric	Shannon's diversity index	SHDI

### 2.6 Statistical analysis

Considering the non‐normal distribution of the data, we performed the multiple‐comparison test after the Kruskal–Wallis test to search for differences in SR, PD, MPD, MNTD, FRic, and FDis for the entire community (Giraudoux et al., 2018). We used the one‐sample t test to determine whether SES was significantly different from 0. The level of statistical significance was set to 0.05.

**FIGURE A2:**
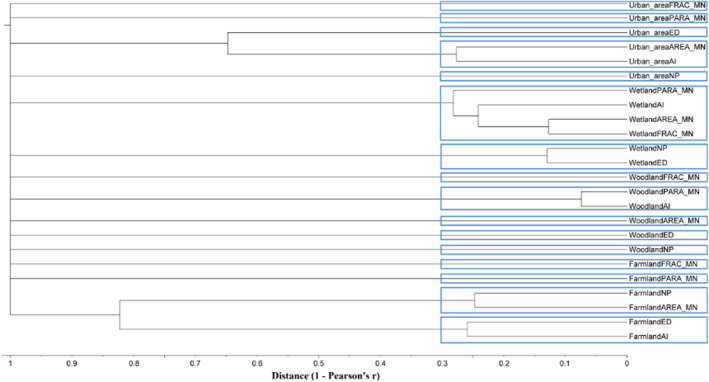
The clustering of landscape metrics at class level for Pearson's correlation. The dendrogram was constructed based on the distance (1—Pearson's *r*).

**FIGURE A3:**
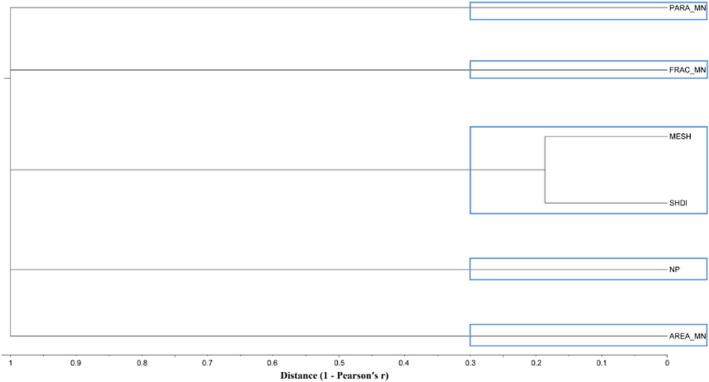
The clustering of landscape metrics at landscape level for Pearson's correlation. The dendrogram was constructed based on the distance (1—Pearson's *r*).

To evaluate the effects of landscape attributes on bird diversity, we first removed outliers from original data and executed missing values imputation with random forest regression (Xia et al., 2017; Zuur et al., 2010). We then used Z‐score transformation to standardize the original data. We calculated Pearson correlation coefficient (*r*) to check the pairwise correlations between the predictor variables (Dormann et al., 2013). Then, we constructed the dendrogram for landscape metrics based on the distance (1 – Pearson's *r*), thereby selecting the metrics with the value of |*r*| to be <0.70 (i.e., selecting one metric in a clade; Figures A2, A3). Then, we built a set of multiple linear regression models by combining the variables retained and used the corrected Akaike information criterion (AICc) to rank models. We performed normality tests on the regression residuals to make sure these multiple linear regression models were robust. Variables selection was based on the models with ΔAICc < 2. We then reconstructed candidate models with variables selected. Given that Akaike weight (*w*_*i*_) indicated that no model was obviously the best (*w*_*i*_ > 0.9; Anderson et al., 2001), we used the model average method to calculate the relative importance (*w*_+_), averaged parameter estimates, and standard errors of variables selected. Model average was carried out with R package “MuMIn” (Bartoń, 2020). All statistical analyses were performed in R 4.0.1 (R Core Team, 2020).

## 3 RESULTS

**FIGURE 2:**
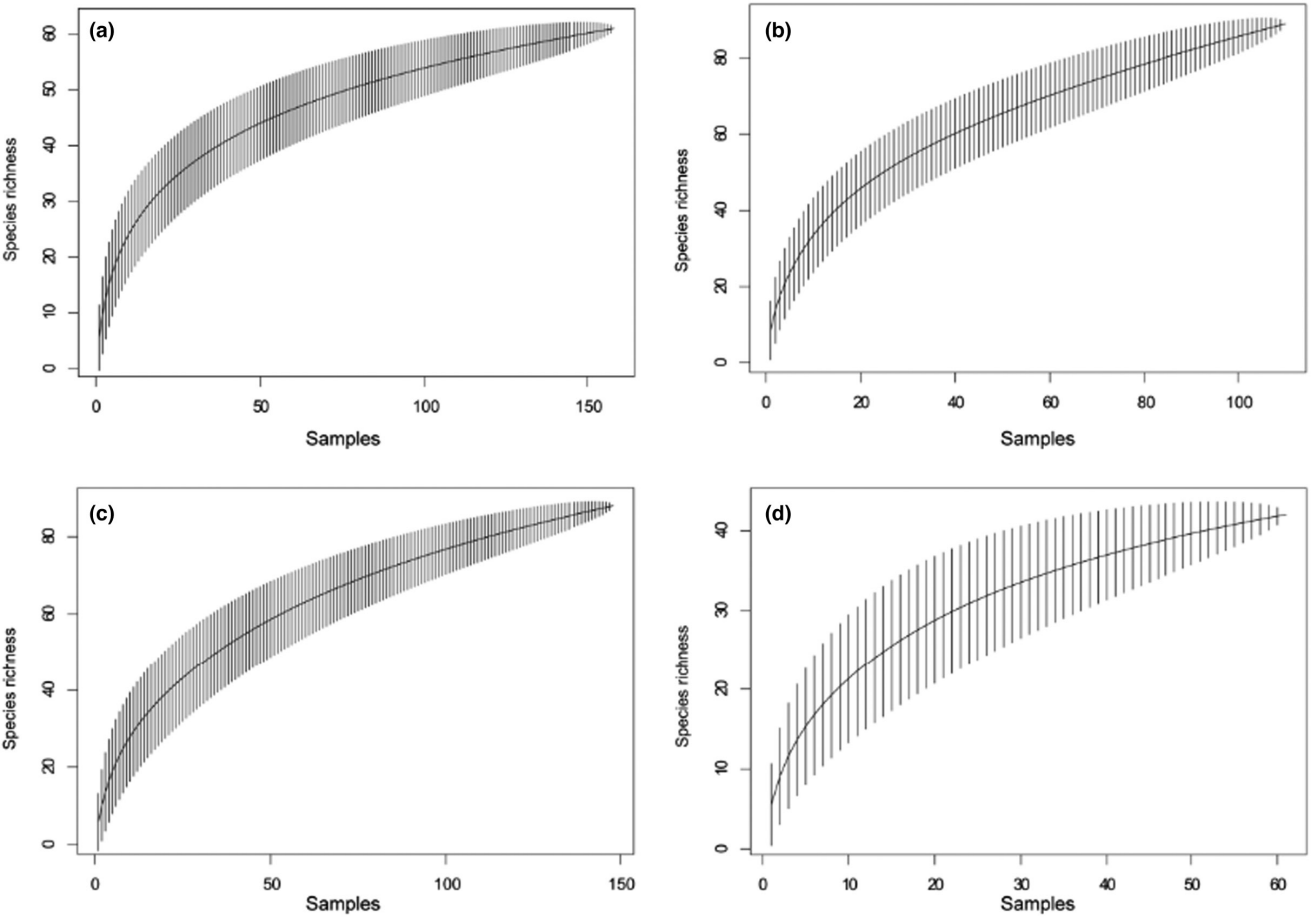
Species accumulation curves based on the samples of survey in different matrix types. The solid lines are the mean species accumulation curve (SAC), with shade areas representing their standard deviation from random permutations of the data. (a) Farmlands; (b) woodlands; (c) wetlands; and (d) urban areas.

After 2 years of investigation, we acquired 453 samples that could be used for subsequent analysis. Species accumulation curves rose smoothly, indicating that we sampled adequately for the four matrix types (Figure 2). From November 2017 to October 2019, 149 bird species were detected at Nanjing Lukou International Airport (Table A1). Eurasian tree sparrow (*Passer montanus*), white‐cheeked starling (*Sturnus cineraceus*), and crested myna (*Acridotheres cristatellus*) were the most common species. Rustic bunting (*Emberiza rustica*) was the vulnerable (VU) species in the IUCN Red List of Threatened Species. Northern lapwing (*Vanellus vanellus*), Japanese quail (*Coturnix japonica*), and reed parrotbill (*Paradoxornis heudei*) were the Near‐Threatened species.

**FIGURE 3:**
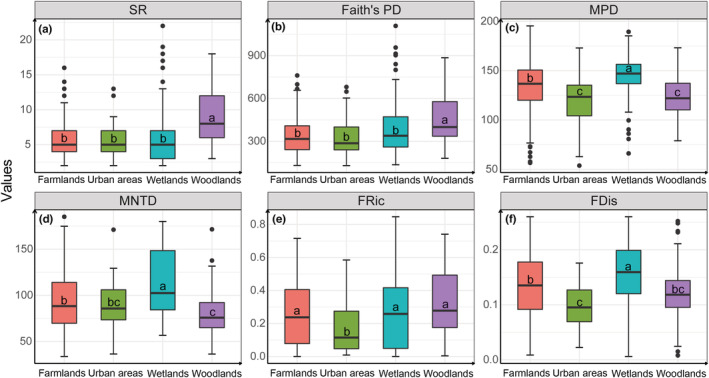
Biodiversity metrics of bird communities in different habitats at Nanjing Lukou International Airport (NLIA). Boxplots with the same letter mean no significant difference after Kruskal‐Wallis test with the significance level set at α = 0.05. (a) SR: species richness; (b) Faith's PD: Faith's phylogenetic distance; (c) MPD: mean pairwise distance; (d) MNTD: mean nearest‐taxon distance; (e) FRic: functional richness; and (f) FDis: functional dispersion.

Species richness and Faith's PD were significantly higher in woodlands than in other matrix types (*χ* ^2^ = 56.528, *p* < 0.001; *χ* ^2^ = 36.484, *p* < 0.001). MPD was highest in wetlands, and MPD was significantly higher in farmlands than in urban areas and woodlands (*χ* ^2^ = 84.617, *p* < 0.001). MNTD was highest in wetlands, while MNTD was significantly higher in farmlands than in woodlands (*χ* ^2^ = 61.747, *p* < 0.001). No significant difference was found in MNTD between farmlands and urban areas and between urban areas and woodlands. FRic was significantly lower in urban areas than in other matrix types (*χ* ^2^ = 19.156, *p* < 0.001). FDis was highest in wetlands, while FDis was significantly higher in farmlands than in urban areas (*χ* ^2^ = 56.328, *p* < 0.001). No significant difference was found in FDis between farmlands and woodlands and between woodlands and urban areas (Figure 3).

**FIGURE 4:**
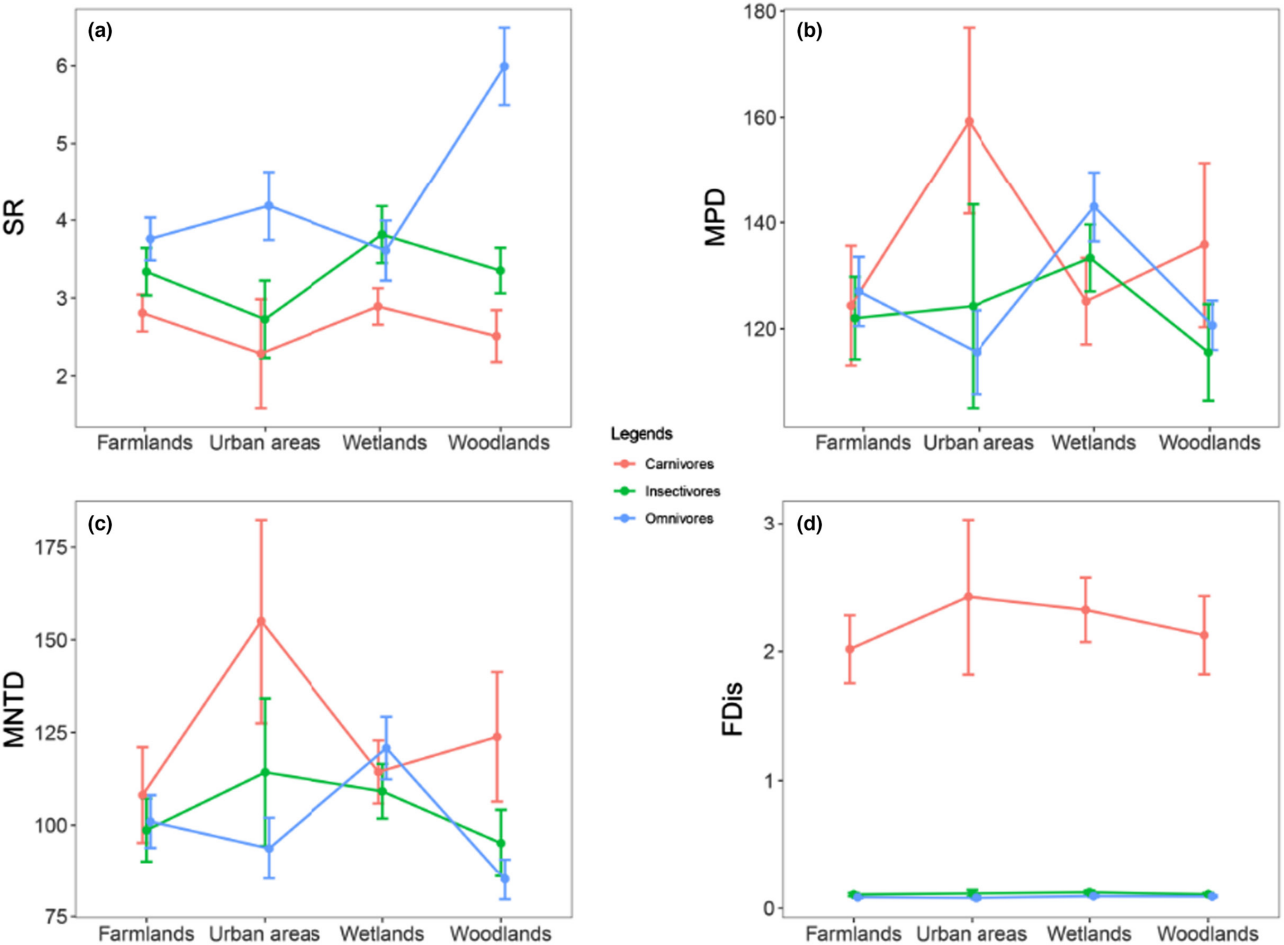
Biodiversity metrics of functional groups in different habitats at Nanjing Lukou International Airport (NLIA). Results are displayed by mean ± 95% CI (confidence interval). (a) SR: species richness; (b) MPD: mean pairwise distance; (c) MNTD: mean nearest‐taxon distance; and (d) FDis: functional dispersion.

Species richness of omnivorous birds was higher than insectivorous and carnivorous birds in all matrix types. However, changes in phylogenetic and functional structure were asynchronous. FDis of carnivorous birds was significantly higher than omnivorous and carnivorous birds while there were no significant differences between MPD and MNTD of functional groups (Figure 4).

**FIGURE 5:**
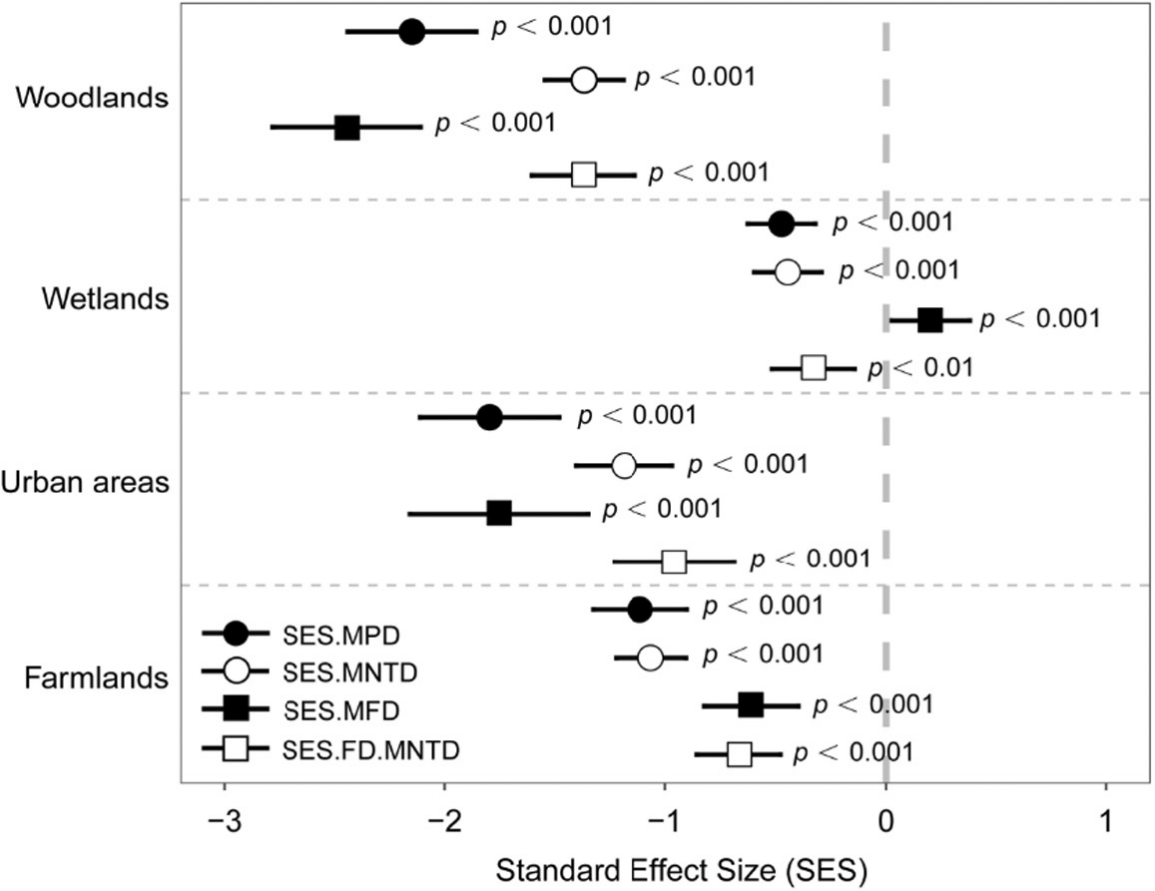
Standardized effect sizes (SES) of phylogenetic and functional diversity and their 95% confidence intervals (with *p*‐values of one‐sample t tests) of bird communities in different matrix types at Nanjing Lukou International Airport (NLIA). SES.MPD is the standardized effect size of mean pairwise distance. SES.MNTD is the standardized effect size of mean nearest‐taxon distance. SES.MFD is the standardized effect size of mean pairwise functional distance. SES.FD.MNTD is the standardized effect size of mean functional nearest‐taxon distance. The gray dash line indicates that the SES value is equal to 0, which is the dividing line to determine whether the community structure is clustered or over‐dispersed.

Phylogenetic signals were significantly related to most traits, indicating strong phylogenetic niche conservatism (Table A2). SES.MPD, SES.MNTD, SES.MFD, and SES.FD.MNTD were significantly different from 0 (*p* < 0.01), indicating phylogenetic and functional clustering in all landscape matrix types (Figure 5).

The results of model average showed that landscape attributes of different matrix types have significant effects on bird diversity, with PARA‐MN of woodlands, ED of farmlands, FRAC‐MN of farmlands, PARA‐MN of urban areas, and AREA‐MN of woodlands being the relatively important predictors (Table 2). Moreover, AREA‐MN‐l was the most important predictors of SR (*w*_+_ = 1.00, *p* < 0.01) and Faith's PD (*w*_+_ = 1.00, *p* < 0.01). MNTD was interpreted based on AREA‐MN‐l (*w*_+_ = 1.00, *p* < 0.01) and NP‐l (*w*_+_ = 1.00, *p* < 0.001). SHDI (*w*_+_ = 1.00, *p* < 0.001) was the most important predictors of FDis (Table 3).

**TABLE 2:** Results generated by model average showing effects of class level of landscape attributes.

Metrics (Z‐score)	Selected variables (Z‐score)	*w*_+_	Estimate	SE	*Z* value
SR	(Intercept)	/	−1.18 × 10^−10^	0.07	0.00
	ED of farmlands	1.00	−0.34	0.11	3.17**
	FRAC‐MN of farmlands	1.00	0.38	0.11	3.36***
	AREA‐MN of urban areas	1.00	0.28	0.11	2.52*
	PARA‐MN of woodlands	1.00	−0.40	0.17	2.31*
Faith's PD	(Intercept)	/	−3.80 × 10^−11^	0.07	0.00
	ED of farmlands	1.00	−0.38	0.11	3.54***
	FRAC‐MN of farmlands	1.00	0.38	0.11	3.41***
	AREA‐MN of urban areas	0.94	0.26	0.12	2.15*
	PARA‐MN of woodlands	0.97	−0.39	0.17	2.27*
MPD	(Intercept)	/	−9.54 × 10^−11^	0.06	0.00
	PARA‐MN of woodlands	1.00	0.23	0.08	2.94**
	ED of urban areas	1.00	−0.34	0.11	3.14**
	NP of urban areas	1.00	0.20	0.08	2.49*
	PARA‐MN of urban areas	1.00	−0.32	0.09	3.55***
	NP of wetlands	1.00	0.23	0.07	3.14**
	AREA‐MN of woodlands	1.00	−0.32	0.09	3.69***
	ED of woodlands	1.00	0.28	0.09	3.20**
MNTD	(Intercept)	/	−4.31 × 10^−12^	0.07	0.00
	FRAC‐MN of woodlands	1.00	−0.26	0.09	3.04**
	NP of wetlands	1.00	0.20	0.07	2.84**
	ED of woodlands	1.00	0.28	0.09	3.00**
FRic	(Intercept)	/	−8.62 × 10^−11^	0.07	0.00
	ED of farmlands	1.00	−0.21	0.09	2.36*
	FRAC‐MN of farmlands	1.00	0.32	0.10	3.14**
	AREA‐MN of woodlands	1.00	−0.39	0.14	2.72**
	PARA‐MN of woodlands	1.00	−0.36	0.14	2.49*
	PARA‐MN of urban areas	0.97	−0.22	0.11	2.04*
FDis	(Intercept)	/	−6.49 × 10^−11^	0.06	0.00
	NP of urban areas	1.00	0.32	0.07	4.34***
	PARA‐MN of urban areas	1.00	−0.41	0.11	3.52***
	AREA‐MN of woodlands	1.00	−0.37	0.10	3.56***

*Note*: Only explanatory variables with significant effects were listed here. The abbreviations are explained as follows: ED, edge density; FRAC‐MN, mean fractal dimension index; AREA‐MN, mean of patch area; PARA‐MN, mean perimeter–area ratio; and NP, number of patches.

*p < 0.05; **p < 0.01; ***p < 0.001.

**TABLE 3:** Results generated by model average showing effects of landscape level of landscape attributes

Metrics (Z‐score)	Selected variables (Z‐score)	*w*_+_	Estimate	SE	*Z* value
SR	(Intercept)	/	−0.39	0.16	2.51*
	AREA‐MN‐l	1.00	0.58	0.22	2.66**
Faith's PD	(Intercept)	/	−0.44	0.16	2.75**
	AREA‐MN‐l	1.00	0.47	0.18	2.58**
MPD	(Intercept)	/	−0.44	0.18	2.43*
MNTD	(Intercept)	/	−0.30	0.18	1.72
	AREA‐MN‐l	1.00	−0.50	0.19	2.68**
	NP‐l	1.00	0.72	0.18	4.00***
FRic	(Intercept)	/	−0.21	0.17	1.25
FDis	(Intercept)	/	−0.47	0.14	3.26**
	SHDI	1.00	0.48	0.14	3.41***

*Note*: Only explanatory variables with significant effects were listed here. The abbreviations are explained as follows: AREA‐MN‐l, mean of patch area; NP‐l, number of patches; and SHDI, Shannon's diversity index.

*p < 0.05; **p < 0.01; ***p < 0.001.

## 4 DISCUSSION

A better understanding of bird community and its drivers is necessary for sustainable urban planning and bird conservation in the areas surrounding airports (Aronson et al., 2014; Hu et al., 2020). It has been proved that bird community composition is directly linked to the probability of bird strikes (Blackwell et al., 2013), emphasizing the need of studying bird community in areas surrounding airports. In this study, we find that landscape matrix types play an important role in shaping bird communities. At Nanjing Lukou International Airport, species richness is highest in woodlands, indicating that woodlands near airports may support more bird species (Figure 3). Woodlands represent hotspots of urban biodiversity, and bird species richness is supported by tree cover (Ferenc et al., 2014). Green space areas can increase species richness in human‐modified landscapes (Zhang et al., 2021). Moreover, PARA‐MN of woodlands has negative effects on species richness, indicating that fragmented woodlands are less suitable for birds (Table 2). Our results show that patchy and regular farmlands also have negative effects on species richness. The intensification of agriculture does have an impact on bird diversity.

Landscape matrix types play an important role in bird phylogenetic and functional diversity (Figure 3). Woodlands are more stable habitats which favor the colonization and successful establishment of many genetic clades (García‐Navas & Thuiller, 2020), thus bird assemblages in woodlands exhibit the highest phylogenetic richness. Based on niche theory, the establishment of bird species might be facilitated by the availability of niches (Sayol et al., 2021). High levels of functional richness illustrate that more functional niches are occupied in woodlands, wetlands, and farmlands. Although more genetic clades can be detected in woodlands, species are less closely related compared to bird assemblages in wetlands, indicating that woodlands contain more evolutionarily unique species (Mestre et al., 2020). The highest FDis of bird assemblages shows that resources in wetlands are sufficiently used by functionally distinct species. In farmlands and urban areas, low phylogenetic and functional diversity may be related to human disturbance and urbanization (Beninde et al., 2015).

Identification and analysis of ecological groups have been fundamental to understanding the entire community structure (Sohil & Sharma, 2020). In this study, we found that the species richness of omnivores is the highest, then insectivores and carnivores at the NLIA. However, changes in phylogenetic and functional structure of functional groups are asynchronous (Figure 4). We can infer that carnivorous birds are more related in lineages than insectivorous and omnivorous birds. Functional traits related to feeding behaviors and foraging strata can reflect the situation of food resources occupation and trophic niche (Si et al., 2017). The analysis of functional structure reveals that carnivores distribute more uniformly in the functional space than insectivores and carnivores (Figure 4d), which indicates that carnivorous birds may occupy more food resources and niches at the NLIA.

The entire community exhibits phylogenetic and functional clustering in all landscape matrix types (Figure 5), which implies environmental filtering governs community assembly at the NLIA (García‐Navas & Thuiller, 2020). The process of bird community assembly in small scales may be dominated by competitive exclusion because limited resources are available (Gómez et al., 2010). A study has found that environmental filtering results in marked shifts in the composition of communities because local and regional habitat structure and function may be changed by urbanization, excluding non‐synanthropic species (Evans et al., 2018). It has been proved that urbanization acts as an environmental filter that governs the assembly of bird community (Schütz & Schulze, 2015). We think that the situation at the NLIA may be the same. In human‐dominated landscapes, local habitat structure is supposed to change due to human disturbance. The abiotic factors function as environmental filters where particular species are selected as a result of their capacity to survive and persist in a given space, resulting in clustered patterns of phylogenetic and functional structure (Adorno et al., 2021; Kraft et al., 2015). We also find that woodlands have the strongest filtering effects, which reinforces the role of woodlands in shaping bird communities.

Patchy and regular farmlands have negative effects on phylogenetic and functional richness of bird communities (Table 2). Evolutionary history is lost because evolutionarily distinct species are more likely to be extinct in farmlands (Frishkoff et al., 2014). We find that the number of phylogenetically related species increases in patchy and fragmented urban areas, indicating the loss of evolutionarily unique branches (Table 2). Moreover, with the developing fragmentation of urban areas, the functional richness of bird communities decreases and the functional structure turns to be simplified, indicating the loss of functionally distinct birds. Urbanization in the areas surrounding airports can cause simplification of bird community structures. The situation of bird communities in woodlands is similar to that in urban areas. Phylogenetic richness and structures of bird communities respond negatively to the fragmentation of woodlands, indicating the loss of evolutionarily unique branches in woodlands. We also find that functional richness of bird communities decreases in fragmented woodlands and the functional structure turns to be simplified with the decrease in areas of woodlands (Table 2). This phenomenon can be interpreted by the shape complexity of woodlands and urban areas. Complex shape may enrich edge structure that has a major impact on birds' selection of vegetation and food resources (Melin et al., 2018). The landscape attributes of wetlands have little effect on bird communities. Only the number of patches of wetlands influences the phylogenetic distance and numbers of phylogenetically related species.

At landscape level, the mean of patch area has the strongest explanatory effects. Species richness and phylogenetic richness respond positively to the mean of patch area (Table 3), indicating that more distantly related birds can be observed with the increase in the mean of patch area. Many studies have proven that landscape diversity reflects landscape heterogeneity level and bird diversity responds positively to landscape heterogeneity (Katayama et al., 2014; Redlich et al., 2018). We find landscape diversity affects bird functional structure positively at the NLIA (Table 3). High landscape heterogeneity near airports means more niches and natural resources are available (Pfeiffer et al., 2018), thus making birds' functional structure complicated.

In this study, 149 bird species were detected at Nanjing Lukou International Airport. Based on the land‐cover data, the landscape was divided into four main types, including farmlands, woodlands, wetlands, and urban areas. Bird species richness, and phylogenetic and functional diversity were strongly affected by landscape matrix types. Species richness and Faith's phylogenetic distance were highest in woodlands, while mean pairwise distance (MPD), mean nearest‐taxon distance (MNTD), and functional dispersion (FDis) were highest in wetlands. Based on the feeding behavior, carnivorous birds had the lowest species richness, but had the highest FDis, which implied that carnivorous birds occupied most niches at the NLIA. This study suggests that functional groups affect the structure of bird communities in different matrix types. We also find that the landscape surrounding the NLIA acts as an environmental filter that governs the bird community assembly, and landscape attributes of different matrix types affect bird diversity. Our results suggest that landscape management surrounding airports can provide new approaches for policymakers to mitigate wildlife strikes.

## AUTHOR CONTRIBUTIONS

**Sijia Yuan:** Data curation (equal); formal analysis (equal); writing – original draft (equal). **Keer Miao:** Data curation (equal); formal analysis (equal); software (equal); writing – original draft (equal). **Ruen Qian:** Investigation (equal); resources (equal); software (equal). **Yang Zhao:** Resources (equal). **Dongfang Hu:** Data curation (equal); formal analysis (equal); resources (equal). **Chaochao Hu:** Conceptualization (lead); data curation (equal); formal analysis (equal); investigation (equal); methodology (equal). **Qing Chang:** Funding acquisition (equal); investigation (equal); writing – original draft (equal).

## FUNDING INFORMATION

This research was funded by the Natural Science Research of Jiangsu Higher Education Institutions of China (grant number 20KJD180004) and Jiangsu Agricultural Biodiversity Cultivation and Utilization Research Center (grant number 100605‐2022‐KY‐00210). The funders had no role in study design, data collection and analysis, decision to publish, or preparation of the manuscript.

## CONFLICT OF INTEREST

The authors declare that they have no conflict of interest.

## Data Availability

Observation data are deposited on Dryad (https://doi.org/10.5061/dryad.mpg4f4r35).

## APPENDIX A

**TABLE A1:** A checklist on birds and different bird groups at Nanjing Lukou International Airport (NLIA).

English name	Scientific name	Functional groups	Total abundance	IUCN
Northern Goshawk	*Accipiter gentilis*	Carnivore	1	LC
Eurasian Sparrowhawk	*Accipiter nisus*	Carnivore	1	LC
Chinese Goshawk	*Accipiter soloensis*	Carnivore	1	LC
Crested Myna	*Acridotheres cristatellus*	Omnivore	2487	LC
Black‐browed Reed Warbler	*Acrocephalus bistrigiceps*	Insectivore	2	LC
Common Sandpiper	*Actitis hypoleucos*	Insectivore	6	LC
Long‐tailed Tit	*Aegithalos caudatus*	Insectivore	189	LC
Eurasian Skylark	*Alauda arvensis*	Omnivore	1097	LC
Oriental Skylark	*Alauda gulgula*	Omnivore	275	LC
Common Kingfisher	*Alcedo atthis*	Piscivore	6	LC
Brown Crake	*Amaurornis akool*	Omnivore	19	LC
White‐breasted Waterhen	*Amaurornis phoenicurus*	Omnivore	1	LC
Common Teal	*Anas crecca*	Omnivore	48	LC
Mallard	*Anas platyrhynchos*	Omnivore	27	LC
Spot‐billed Duck	*Anas poecilorhyncha*	Omnivore	101	LC
Olive‐backed Pipit	*Anthus hodgsoni*	Insectivore	335	LC
Richard's Pipit	*Anthus richardi*	Insectivore	12	LC
Paddyfield Pipit	*Anthus rufulus*	Insectivore	2	LC
Gray Heron	*Ardea cinerea*	Piscivore	4	LC
Purple Heron	*Ardea purpurea*	Carnivore, Insectivore	1	LC
Chinese Pond heron	*Ardeola bacchus*	Carnivore, Insectivore	215	LC
Short‐eared Owl	*Asio flammeus*	Carnivore	2	LC
Long‐eared Owl	*Asio otus*	Carnivore	1	LC
Little Owl	*Athene noctua*	Carnivore	2	LC
Chinese Bamboo Partridge	*Bambusicola thoracicus*	Omnivore	29	LC
Cattle Egret	*Bubulcus ibis*	Carnivore, Insectivore	852	LC
Common Buzzard	*Buteo buteo*	Carnivore	2	LC
Striated Heron	*Butorides striata*	Carnivore, Insectivore	2	LC
Long‐toed Stint	*Calidris subminuta*	Insectivore	2	LC
Gray Nightjar	*Caprimulgus indicus*	Insectivore	1	LC
Gray‐capped Greenfinch	*Carduelis sinica*	Granivore	162	LC
Eurasian Siskin	*Carduelis spinus*	Omnivore	9	LC
Great Egret	*Casmerodius albus*	Carnivore, Insectivore	6	LC
Lesser Coucal	*Centropus bengalensis*	Insectivore	3	LC
Pied Kingfisher	*Ceryle rudis*	Piscivore	14	LC
Kentish Plover	*Charadrius alexandrinus*	Carnivore, Insectivore	11	LC
Little Ringed Plover	*Charadrius dubius*	Insectivore	35	LC
Greater Sand Plover	*Charadrius leschenaultii*	Insectivore	2	LC
Long‐billed Plover	*Charadrius placidus*	Omnivore	20	LC
Oriental Plover	*Charadrius veredus*	Insectivore	4	LC
Whiskered Tern	*Chlidonias hybrida*	Carnivore, Insectivore	1	LC
Zitting Cisticola	*Cisticola juncidis*	Omnivore	3	LC
Rock Pigeon	*Columba livia*	Granivore	246	LC
Japanese Quail	*Coturnix japonica*	Omnivore	1	NT
Common Cuckoo	*Cuculus canorus*	Insectivore	2	LC
Indian Cuckoo	*Cuculus micropterus*	Insectivore	3	LC
Lesser Cuckoo	*Cuculus poliocephalus*	Insectivore	1	LC
Himalayan Cuckoo	*Cuculus saturatus*	Insectivore	1	LC
Large Hawk‐cuckoo	*Cuculus sparverioides*	Insectivore	1	LC
Azure‐winged Magpie	*Cyanopica cyanus*	Omnivore	175	LC
Gray Treepie	*Dendrocitta formosae*	Omnivore	1	LC
Gray‐capped Woodpecker	*Dendrocopos canicapillus*	Insectivore	1	LC
Great Spotted Woodpecker	*Dendrocopos major*	Insectivore	1	LC
Black Drongo	*Dicrurus macrocercus*	Insectivore	124	LC
Little Egret	*Egretta garzetta*	Carnivore, Insectivore	985	LC
Black‐winged Kite	*Elanus caeruleus*	Carnivore	1	LC
Yellow‐browed Bunting	*Emberiza chrysophrys*	Omnivore	3	LC
Yellow‐throated Bunting	*Emberiza elegans*	Omnivore	24	LC
Chestnut‐eared Bunting	*Emberiza fucata*	Omnivore	3	LC
Pallas's Bunting	*Emberiza pallasi*	Omnivore	1	LC
Little Bunting	*Emberiza pusilla*	Omnivore	19	LC
Rustic Bunting	*Emberiza rustica*	Omnivore	8	VU
Reed Bunting	*Emberiza schoeniclus*	Omnivore	1	LC
Black‐faced Bunting	*Emberiza spodocephala*	Omnivore	196	LC
Tristram's Bunting	*Emberiza tristrami*	Omnivore	2	LC
Yellow‐billed Grosbeak	*Eophona migratoria*	Omnivore	313	LC
Japanese Grosbeak	*Eophona personata*	Omnivore	1	LC
Asian Koel	*Eudynamys scolopaceus*	Omnivore	3	LC
Amur Falcon	*Falco amurensis*	Carnivore	73	LC
Merlin	*Falco columbarius*	Carnivore	4	LC
Peregrine Falcon	*Falco peregrinus*	Carnivore	1	LC
Eurasian Hobby	*Falco subbuteo*	Carnivore	3	LC
Common Kestrel	*Falco tinnunculus*	Carnivore	14	LC
Yellow‐rumped Flycatcher	*Ficedula zanthopygia*	Insectivore	1	LC
Brambling	*Fringilla montifringilla*	Omnivore	490	LC
Common Coot	*Fulica atra*	Omnivore	4	LC
Common Snipe	*Gallinago gallinago*	Insectivore	10	LC
Swinhoe's Snipe	*Gallinago megala*	Insectivore	1	LC
Pintail Snipe	*Gallinago stenura*	Insectivore	7	LC
Common Moorhen	*Gallinula chloropus*	Omnivore	614	LC
Masked Laughingthrush	*Garrulax perspicillatus*	Omnivore	193	LC
Oriental Pratincole	*Glareola maldivarum*	Insectivore	49	LC
Red‐rumped Swallow	*Hirundo daurica*	Insectivore	298	LC
Barn Swallow	*Hirundo rustica*	Insectivore	661	LC
Pheasant‐tailed Jacana	*Hydrophasianus chirurgus*	Omnivore	8	LC
Cinnamon Bittern	*Ixobrychus cinnamomeus*	Carnivore, Insectivore	1	LC
Black Bittern	*Ixobrychus flavicollis*	Carnivore, Insectivore	2	LC
Yellow Bittern	*Ixobrychus sinensis*	Carnivore, Insectivore	6	LC
Brown Shrike	*Lanius cristatus*	Carnivore, Insectivore	67	LC
Long‐tailed Shrike	*Lanius schach*	Carnivore, Insectivore	558	LC
Chinese Gray Shrike	*Lanius sphenocercus*	Carnivore, Insectivore	1	LC
White‐rumped Munia	*Lonchura striata*	Omnivore	61	LC
Intermediate Egret	*Mesophoyx intermedia*	Carnivore, Insectivore	18	LC
Black Kite	*Milvus migrans*	Carnivore	8	LC
White Wagtail	*Motacilla alba*	Insectivore	331	LC
Gray Wagtail	*Motacilla cinerea*	Insectivore	10	LC
Yellow Wagtail	*Motacilla flava*	Insectivore	2	LC
Asian Brown Flycatcher	*Muscicapa dauurica*	Insectivore	1	LC
Whimbrel	*Numenius phaeopus*	Insectivore	1	LC
Black‐crowned Night Heron	*Nycticorax nycticorax*	Carnivore, Insectivore	540	LC
Collared Scops Owl	*Otus lettia*	Carnivore	3	LC
Oriental Scops Owl	*Otus sunia*	Carnivore	1	LC
Reed Parrotbill	*Paradoxornis heudei*	Insectivore	1	NT
Vinous‐throated Parrotbill	*Paradoxornis webbianus*	Omnivore	210	LC
Great Tit	*Parus major*	Insectivore	31	LC
Eurasian Tree Sparrow	*Passer montanus*	Omnivore	7812	LC
Common Pheasant	*Phasianus colchicus*	Omnivore	52	LC
Daurian Redstart	*Phoenicurus auroreus*	Insectivore	44	LC
Arctic Warbler	*Phylloscopus borealis*	Insectivore	3	LC
Dusky Warbler	*Phylloscopus fuscatus*	Insectivore	2	LC
Inornate Warbler	*Phylloscopus inornatus*	Insectivore	4	LC
Lemon‐rumped Warbler	*Phylloscopus proregulus*	Insectivore	9	LC
Black‐billed Magpie	*Pica pica*	Omnivore	803	LC
Gray‐faced Woodpecker	*Picus canus*	Insectivore	1	LC
Pacific Golden Plover	*Pluvialis fulva*	Carnivore, Insectivore	5	LC
Gray Plover	*Pluvialis squatarola*	Carnivore, Insectivore	5	LC
Plain Prinia	*Prinia inornata*	Insectivore	92	LC
Light‐vented Bulbul	*Pycnonotus sinensis*	Omnivore	1565	LC
Water Rail	*Rallus aquaticus*	Omnivore	1	LC
Chinese Penduline Tit	*Remiz consobrinus*	Omnivore	25	LC
Greater Painted Snipe	*Rostratula benghalensis*	Omnivore	1	LC
Common Stonechat	*Saxicola torquatus*	Insectivore	2	LC
Eurasian Woodcock	*Scolopax rusticola*	Omnivore	3	LC
Collared Finchbill	*Spizixos semitorques*	Omnivore	28	LC
Spotted Dove	*Stigmatopelia chinensis*	Omnivore	1354	LC
Oriental Turtle Dove	*Streptopelia orientalis*	Omnivore	506	LC
White‐cheeked Starling	*Sturnus cineraceus*	Omnivore	2877	LC
Black‐collared Starling	*Sturnus nigricollis*	Omnivore	36	LC
Red‐billed Starling	*Sturnus sericeus*	Omnivore	218	LC
Common Starling	*Sturnus vulgaris*	Omnivore	3	LC
Little Grebe	*Tachybaptus ruficollis*	Carnivore, Insectivore	255	LC
Orange‐flanked Bush Robin	*Tarsiger cyanurus*	Omnivore	3	LC
Spotted Redshank	*Tringa erythropus*	Insectivore	34	LC
Wood Sandpiper	*Tringa glareola*	Insectivore	3	LC
Common Greenshank	*Tringa nebularia*	Insectivore	6	LC
Green Sandpiper	*Tringa ochropus*	Insectivore	78	LC
Marsh Sandpiper	*Tringa stagnatilis*	Insectivore	3	LC
Common Redshank	*Tringa totanus*	Insectivore	1	LC
Gray‐backed Thrush	*Turdus hortulorum*	Omnivore	44	LC
Eurasian Blackbird	*Turdus merula*	Omnivore	494	LC
Dusky Thrush	*Turdus naumanni*	Omnivore	8	LC
Eyebrowed Thrush	*Turdus obscurus*	Omnivore	7	LC
Pale Thrush	*Turdus pallidus*	Insectivore	1	LC
Yellow‐legged Buttonquail	*Turnix tanki*	Omnivore	2	LC
Eurasian Hoopoe	*Upupa epops*	Insectivore	57	LC
Blue Magpie	*Urocissa erythrorhyncha*	Omnivore	2	LC
Gray‐headed Lapwing	*Vanellus cinereus*	Omnivore	388	LC
Northern Lapwing	*Vanellus vanellus*	Omnivore	83	NT
Eurasian Scaly Thrush	*Zoothera dauma*	Insectivore	1	LC

Abbreviations: LC, least‐concern species; NT, near‐threatened species; and VU, vulnerable species.

**TABLE A2:** Traits used to measure functional diversity and phylogenetic signals of traits.

Trait types	Traits	Value type	Phylogenetic signal	*p*_Brownian_	*p*_random_
Morphological characteristics	Body length	Continuous	*K* = 0.926	‐	0.001
	Bill length	Continuous	*K* = 1.119	‐	0.001
	Wing length	Continuous	*K* = 1.185	‐	0.001
	Tarsus length	Continuous	*K* = 1.030	‐	0.001
	Body mass	Continuous	*K* = 0.267	‐	0.006
Nest sites	Ground	Binary	*D* = −0.278	0.834	0
	Water	Binary	*D* = −0.324	0.673	0.006
	Shrub	Binary	*D* = 0.130	0.413	0.001
	Canopy	Binary	*D* = −0.208	0.749	0
	Wall	Binary	*D* = −0.942	0.847	0.001
Feeding guilds	Carnivores	Binary	*D* = −0.447	0.876	0
	Insectivores	Binary	*D* = −0.349	0.871	0
	Omnivores	Binary	*D* = −0.389	0.884	0
	Granivores	Binary	*D* = −8.535	0.799	0.169
	Piscivores	Binary	*D* = −0.902	0.938	0
Foraging strata	Below water surface	Binary	*D* = 0.780	0.145	0.341
	Ground	Binary	*D* = 0.389	0.114	0
	Understory	Binary	*D* = −0.050	0.593	0
	Mid‐high	Binary	*D* = −0.162	0.710	0

*Note*: Both probabilities resulting from Brownian (*p*_Brownian_) and random (*p*_random_) phylogenetic structures were exhibited.
